# Pressure in isochoric systems containing aqueous solutions at subzero Centigrade temperatures

**DOI:** 10.1371/journal.pone.0183353

**Published:** 2017-08-17

**Authors:** Gideon Ukpai, Gabriel Năstase, Alexandru Șerban, Boris Rubinsky

**Affiliations:** 1 Department of Mechanical Engineering, University of California Berkeley, Berkeley, CA, United States of America; 2 Department of Building Services, Transilvania University of Brașov, BRAȘOV RO, Romania; Genomics Institute of the Novartis Research Foundation, UNITED STATES

## Abstract

**Objective:**

Preservation of biological materials at subzero Centigrade temperatures, cryopreservation, is important for the field of tissue engineering and organ transplantation. Our group is studying the use of isochoric (constant volume) systems of aqueous solution for cryopreservation. Previous studies measured the pressure-temperature relations in aqueous isochoric systems in the temperature range from 0°C to – 20°C. The goal of this study is to expand the pressure-temperature measurement beyond the range reported in previous publications.

**Materials and methods:**

To expand the pressure-temperature measurements beyond the previous range, we have developed a new isochoric device capable of withstanding liquid nitrogen temperatures and pressures of up to 413 MPa. The device is instrumented with a pressure transducer than can monitor and record the pressures in the isochoric chamber in real time. Measurements were made in a temperature range from – 5°C to liquid nitrogen temperatures for various solutions of pure water and Me_2_SO (a chemical additive used for protection of biological materials in a frozen state and for vitrification (glass formation) of biological matter). Undissolved gaseous are is carefully removed from the system.

**Results:**

Temperature-pressure data from – 5°C to liquid nitrogen temperature for pure water and other solutions are presented in this study. Following are examples of some, temperature-pressure values, that were measured in an isochoric system containing pure water: (- 20°C, 187 MPa); (-25°C, 216 MPa); (- 30°C, 242.3 MPa); (-180°C, 124 MPa). The data is consistent with the literature, which reports that the pressure and temperature at the triple point, between ice I, ice III and water is, - 21.993°C and 209.9 MPa, respectively. It was surprising to find that the pressure in the isochoric system increases at temperatures below the triple point and remains high to liquid nitrogen temperatures. Measurements of pressure-temperature relations in solutions of pure water and Me_2_SO in different concentrations show that, for concentrations in which vitrification is predicted, no increase in pressure was measured during rapid cooling to liquid nitrogen temperatures. However, ice formation either during cooling or warming to and from liquid nitrogen temperatures produced an increase in pressure.

**Conclusions:**

The data obtained in this study can be used to aid in the design of isochoric cryopreservation protocols. The results suggest that the pressure measurement is important in the design of “constant volume” systems and can provide a simple means to gain information on the occurrence of vitrification and devitrification during cryopreservation processes of aqueous solutions in an isochoric system.

## Introduction

Advances in tissue engineering and the growing need for organ transplantation have led to an increased interest in developing new technologies for long term preservation of biological materials at cryogenic temperatures, cryopreservation, [1]. The field of cryopreservation has experienced substantial advances during the last century. Luyet was the first to report successful cryopreservation of living biological matter in liquid nitrogen and in liquid air, [2, 3]. He reports the successful preservation by vitrification of frog sperm [2], single cell microorganisms and onion epithelial cells [3], moss [4] and even the resumption of the heartbeat of chick embryos frozen in liquid nitrogen [5]. Luyet’s early cryopreservation research focused on vitrification (glass formation) of water. Vitrification of aqueous solutions was achieved by rapid cooling of small volumes to cryogenic temperatures [6]. In a major breakthrough, Polge, Smith and Parkes [7] found that the addition of certain chemicals to the preservation solution facilitates survival of living biological matter, when frozen with low cooling rates and in larger volumes. Subsequent research in the field of cryopreservation led to a fundamental understanding of the mechanisms of cell death and survival from freezing. This understanding was summarized by Mazur in a seminal, 1970 paper [8]. The paper states that the main mechanisms affecting cryopreservation are: the rate of cooling during freezing, the temperature of preservation and the time of preservation, the rate of warming during thawing, and the concentration and nature of various cryoprotectants. Today, these are still considered the important parameters in designing cryopreservation protocols by freezing. Since the early 1980’s, Fahy and his colleagues, have made major advances in the field of cryopreservation by vitrification and developed new vitrification protocols for cryopreservation using combinations of chemical additives that promote vitrification [9–11],[12]. The literature on cryopreservation is voluminous and numerous reviews and papers are being continuously published, e.g. [13–17]. Currently, there are two main approaches to cryopreservation: freezing with low, optimal cooling rates aided by use of cryoprotective agents [8, 18, 19] and vitrification by either rapid freezing or use of chemicals that promote vitrification [9–11],[12]. To the best of our knowledge, until recently [20], cryopreservation was done in constant pressure (isobaric) systems. In the majority of cryopreservation applications, the constant pressure is atmospheric, e.g. [8, 18, 19]. In a few exceptions the isobaric systems are hyperbaric, e.g. [21, 22]. The use of isochoric systems (constant volume) of aqueous solutions for cryopreservation was proposed first in [20].

To provide context to this study, a brief review of our earlier work follows. Our research began by developing the fundamental theoretical thermodynamic relations between temperature, pressure and composition in aqueous solutions of interest to cryobiology, in a constant volume (isochoric) system [20, 23]. The theoretical results were confirmed experimentally using an isochoric system that we developed for this purpose [24]. The first isochoric device we built was designed to withstand a pressure of 75.8 MPa and temperatures to about– 15°C. The device was instrumented with means to monitor pressure and temperature. The second study, by Szobota, was theoretical and dealt with vitrification of aqueous solutions in an isochoric system [25]. The study predicted that isochoric systems would promote vitrification. Preciado developed a new isochoric device that withstood pressures up to 275 MPa and temperatures down to– 20°C [23, 26, 27]. The device was used to expand the range of experimental measurements of pressure and temperature in an isochoric system of aqueous solutions from– 12°C [24] to– 20°C. Recently, the device described in [23, 26, 27] was used to demonstrate the survival of the nematode *C*. *Elegans* in an isochoric system at a temperature of– 5°C and pressure of 60 MPa [28]. The use of isochoric systems for food preservation was studied in another recent set of publications [29–31]. Last, a recent publication by Perez et al. has shown that the thermodynamic properties of a two phase undissolved gaseous air–water mixture isochoric system, are substantially different from those of a single phase liquid isochoric aqueous system [23]. The paper by Perez et al. is important because it shows that the isochoric systems for cryopreservation discussed in this study as well as in all our previous studies, require the minimization and preferably the avoidance of undissolved gases in the aqueous solutions. Our research is focused on using isochoric systems of aqueous solutions that avoid or minimize the presence of undissolved gases in aqueous solutions.

The goal of the research reported in this paper is to further advance the understanding of the thermodynamics in isochoric (constant volume) aqueous systems of potential interest to cryopreservation at subzero Centigrade temperatures [20, 24]. The study expands the earlier work on isochoric systems of aqueous solutions relevant to cryopreservation, to a wider range of pressures and temperatures. We use a new isochoric chamber designed to withstand pressures up to 413 MPa and liquid nitrogen temperatures. Measurements of temperature and pressure to liquid nitrogen temperatures were performed using this chamber. We report the relation between pressure and temperature in an isochoric system, devoid of air in gaseous form, comprised of solutions of pure water with different concentrations of Me2SO (a chemical commonly used for improving biological matter survival following cryopreservation by freezing and by vitrification).

## Materials and methods

### Isochoric system

**Fig 1:**
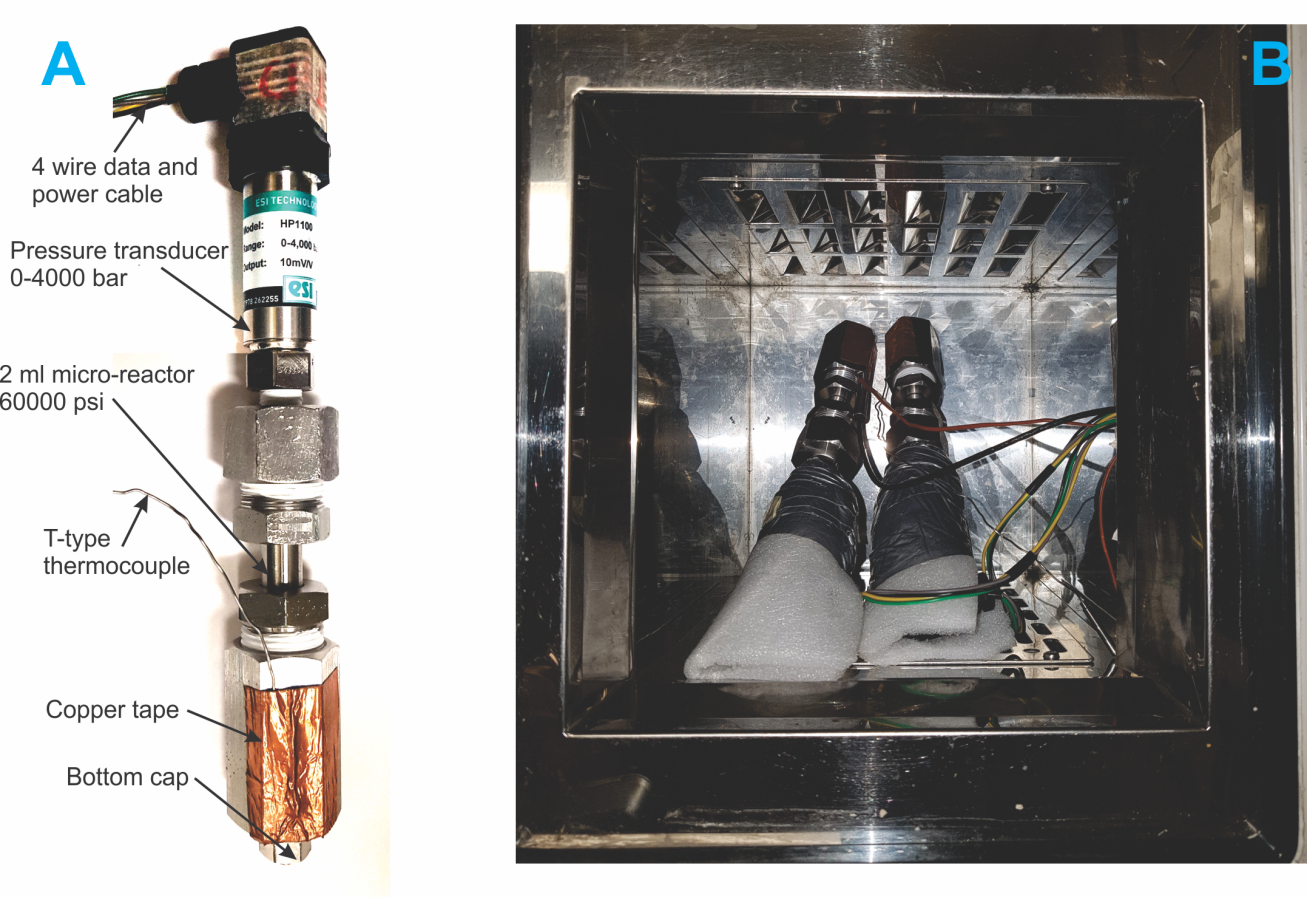
Experimental system. A) a photograph of the isochoric chamber, B) Two isochoric devices in the cooling chamber of a Planer Kryo 10 series III controlled rate freezer.

The isochoric freezing system is a simple constant volume chamber capable of withstanding the pressures that develop in the system, with minimal deformation. The chamber is instrumented with a pressure transducer. A photograph of the system and its components is shown in Fig 1A. The isochoric chamber is a 2 mL 316 stainless steel commercial micro-reactor MS-1 (total inner volume with fittings 3 mL, working pressure 60,000 psi) custom designed by High Pressure Equipment Company (Erie, PA, USA) to withstand the specified pressure and liquid nitrogen temperatures. The MS-1 micro reactor has an inside diameter of 3/16”, an outside diameter of 9/16”, an inside depth of 4” and an overall length of 7”. The isochoric micro-reactor is connected to an ESI Technology Ltd HP1100 0–4000 bar pressure transducer, connected to a DATAQ Instruments Model DI-245 Voltage and Thermocouple DAQ 4 channel data logger connected to a laptop running DATAQ Instruments Hardware Manager. The stored data was viewed and exported from the WinDAQ Waveform browser, installed on a laptop. The pressure transducer is made of a Silicon-on-Sapphire sensor combined with a diaphragm machined from a single piece of titanium alloy. In these experiments, polyethylene pipe insulation fixed with duct tape was used to insulate the pressure transducer and thereby minimize heat flux through the pressure transducer. For safety, the system employs a safety head equipped with a rupture disk to limit the pressure to 60,000 psi (413 MPa). Omega T-type Thermocouples connected to Extech Instruments EasyView™ 15 Thermometer Datalogger were also attached to the outside of the isochoric chambers as a second check to verify the set temperatures. The thermocouples were attached on the outside of the isochoric micro-reactors using copper foil tape with conductive adhesive.

Three different experiments were performed with this setup. In the first experiment, the isochoric system was immersed in a water–ethylene glycol bath (50/50) cooled by means of a NesLab RT-140 cooling system (Thermo Fisher Scientific, Waltham, MA, USA). This device can control temperatures to– 35°C. In the second experiment, the isochoric system was placed in a Planer Kryo 10 series III controlled rate freezer capable of reaching liquid nitrogen temperatures (Fig 1B). In the third experiment, the isochoric system was immersed in a 2L stainless steel Thermo Scientific Thermo-Flask 2123 (Thermo Fisher Scientific) filled with liquid nitrogen at –196°C, to achieve higher cooling rates.

### Materials

For the first and second experiments, we used steam distilled water (Alhambra). For the third experiment, we prepared several solutions of steam distilled water (Alhambra) and Dimethyl Sulfoxide (ME_2_SO with MW:78.13 g/mole, from Thermo Scientific, Prod.#20688) with the desired concentration to be tested, in w/v (g/mL). Each solution is prepared by weighing the desired ME_2_SO mass in a 50 mL volumetric flask and then adding the necessary volume of water to get the desired concentration and mixing. Heat is released when combining the two liquids so the volumetric flask containing the mixed solution is cooled to room temperature in a water bath. More water is usually added to the mixing flask to get the desired volume because the volume typically shrinks when mixed.

### Experimental protocol

For the first experiment, two identical micro-reactors were completely filled with pure distilled water (3 mL), including the pressure transducer cavity, and closed. It is important to emphasize that in our studies care was exercised to eliminate undissolved gaseous air from the system; at the pressures reached in this study, gaseous air is compressible so its presence will affect the results [23]. The two isochoric systems were then immersed in the cooling bath (NesLab) at 0°C. The temperature of the cooling bath was then set at -5°C for 30 minutes after which it was decreased by 5°C and held at -10°C for another 30 minutes. The temperatures were decreased in -5°C decrements down to -30°C and held at each set temperature for 30 minutes. After 30 mins at -30°C, the temperatures were increased in 5°C increments to 0°C and held at each set temperature for 30 minutes. The pressure in the isochoric system was monitored and recorded throughout the experiment. This experiment was repeated three times.

For the second experiment, two identical micro-reactors were filled with pure distilled water (3 mL), including the pressure transducer cavity, and closed. As previously stated, it is important to emphasize that care must be exercised to eliminate undissolved gaseous air from the system [23]. The two isochoric systems were then placed in the controlled rate freezer (Planer). The freezer was used to cool and heat the chambers following a pre-programmed temperature profile. The chambers were cooled from 20°C to -40°C at a rate of -20°C/min and held at -40°C for 30 minutes. Next, the chambers were cooled in -40°C decrements down to -160°C at a rate of -20°C/min and held at each temperature for 30 mins. After which the chambers were cooled from -160°C to -180°C at a rate of -20°C/min and held at -180°C for 30 minutes. After 30 mins at -180°C, the chambers were warmed in reverse manner to the cooling. From -180˚C the chambers were heated to -160°C at 20°C/min and held at -160°C for 30 minutes. The chambers were then heated in 40°C increments up to -40°C at a rate of 20°C/min and held at each temperature for 30 min. From -40°C the chambers were then warmed to 20°C at a rate of 20°C/min at which point the program stopped. The pressures were recorded throughout the experiment. This experiment was repeated three times.

For the third experiment, before filling the micro-reactor with a new solution, the micro-reactor and all its components are flushed with water and cleaned with paper towels. For each concentration of water-ME_2_SO mixture, the micro-reactor was filled completely (3 mL), including the pressure transducer cavity, with the solution and closed. Again, care was exercised to eliminate undissolved air from the system, [23]. The isochoric system containing the solution was then immersed in the Thermo-Flask (Thermo Scientific) filled with liquid nitrogen, for 15 minutes. Additional liquid nitrogen was added to the flask to ensure the chamber was fully immersed in liquid nitrogen over the entire 15-minute period. The cooling rate measured by the thermocouple on the isochoric chamber was 53.2°C / min. After 15 minutes in liquid nitrogen, the isochoric system was removed and warmed in air for 45 minutes or the time it took for the pressure in the chamber to return to its initial value at room temperature. The pressures were recorded while the device was immersed in liquid nitrogen and while it was being warmed. This experiment was repeated for several concentrations of Me_2_SO in pure water. For each solution, the experiment was repeated three times. In the end, the recorded data was exported to Microsoft Excel for post-processing.

Each of the three different experiments described above was done in triplicate.

## Results and discussion

**Fig 2:**
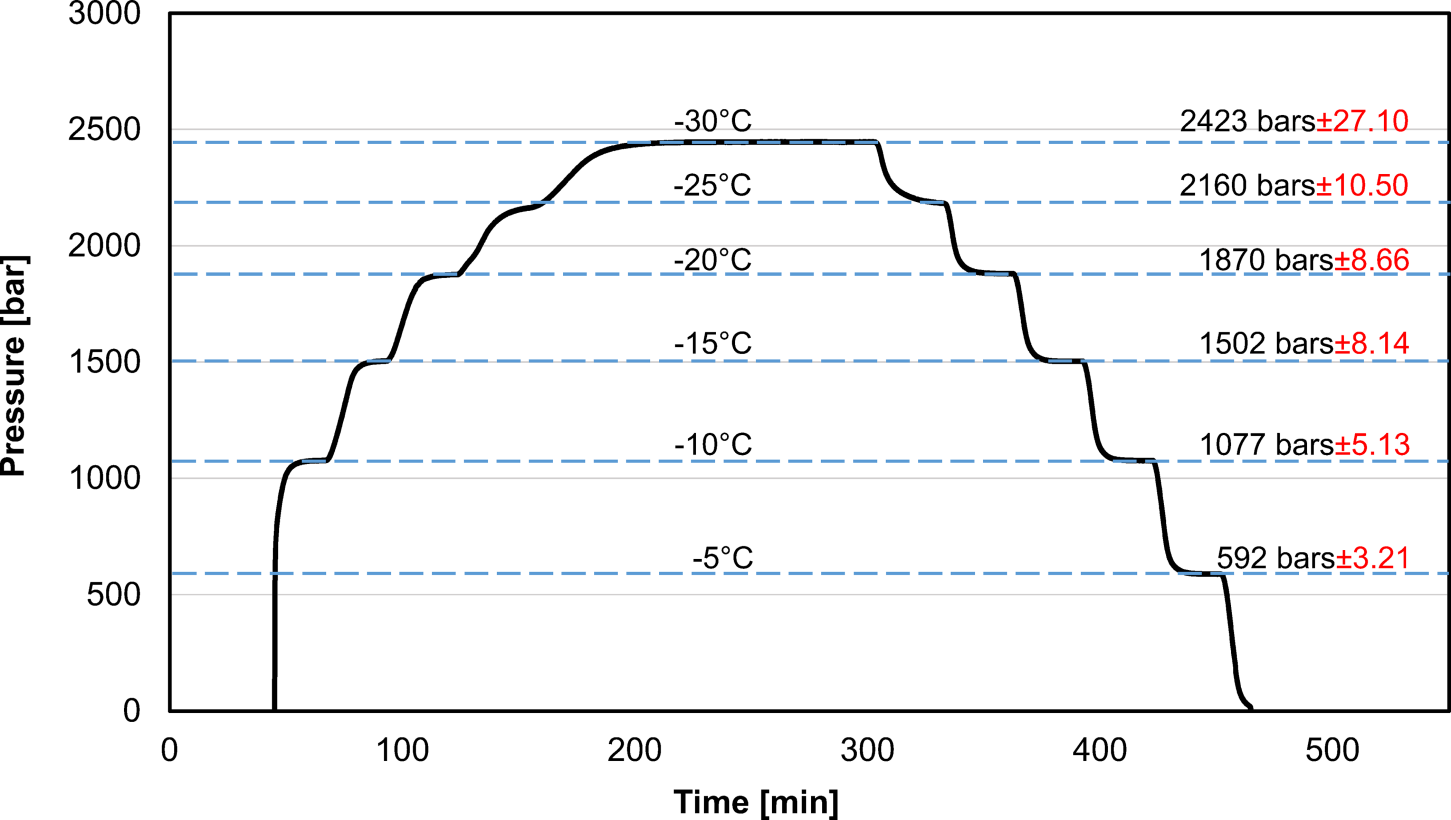
Pressure as a function of time during cooling and warming. The experiments were performed by setting a constant temperature for periods of 30 minutes during cooling and warming. The constant temperatures and their corresponding steady state pressure (with the standard deviation) measured are listed on the figure. Supporting information contained in S1 File

Fig 2 shows typical results obtained from the first set of experiments. These results are typical to all three replicates. Fig 2 displays pressure as a function of time throughout the experiment. The temperature was changed stepwise and the constant temperature values used in this experiment are listed on the horizontal lines in the figure. Each temperature was kept constant for 30 minutes, during cooling and heating, except for the– 30°C case, where the temperature was kept constant for 120 minutes. The results should be read as follows: the intersection between the pressure-time curve and the horizontal temperature lines delineates regions of constant temperatures. For example, during cooling, the part of the pressure-time curve between the– 10°C and the– 15°C horizontal lines, represents the pressure temperature correlation for the period when the temperature of the bath was lowered from– 10°C to– 15°C. However, during heating, the part of the pressure-time curve between the– 10°C and the– 15°C, is for the period in which the temperature of the bath was increased from– 15°C to– 10°C. The figure also lists, next to the constant temperature line, the pressures recorded at that temperature at steady state and the standard deviation from six measurements–three during cooling and three during warming.

Several interesting observations emerge from this figure. First, it is evident that steady state values of pressure are achieved after the 30 minutes at any given constant temperature. The other interesting observation is that the same values of pressure were obtained at the specified constant temperatures both while cooling and while heating to those temperatures, i.e. there was no hysteresis. This suggests that, in this study, the measured values of pressure and temperature were at thermodynamic equilibrium. (As a side note, there is no data point for – 5°C during freezing. This is because the system supercooled and freezing by random nucleation occurred only at a temperature lower than– 5°C.)

**Fig 3:**
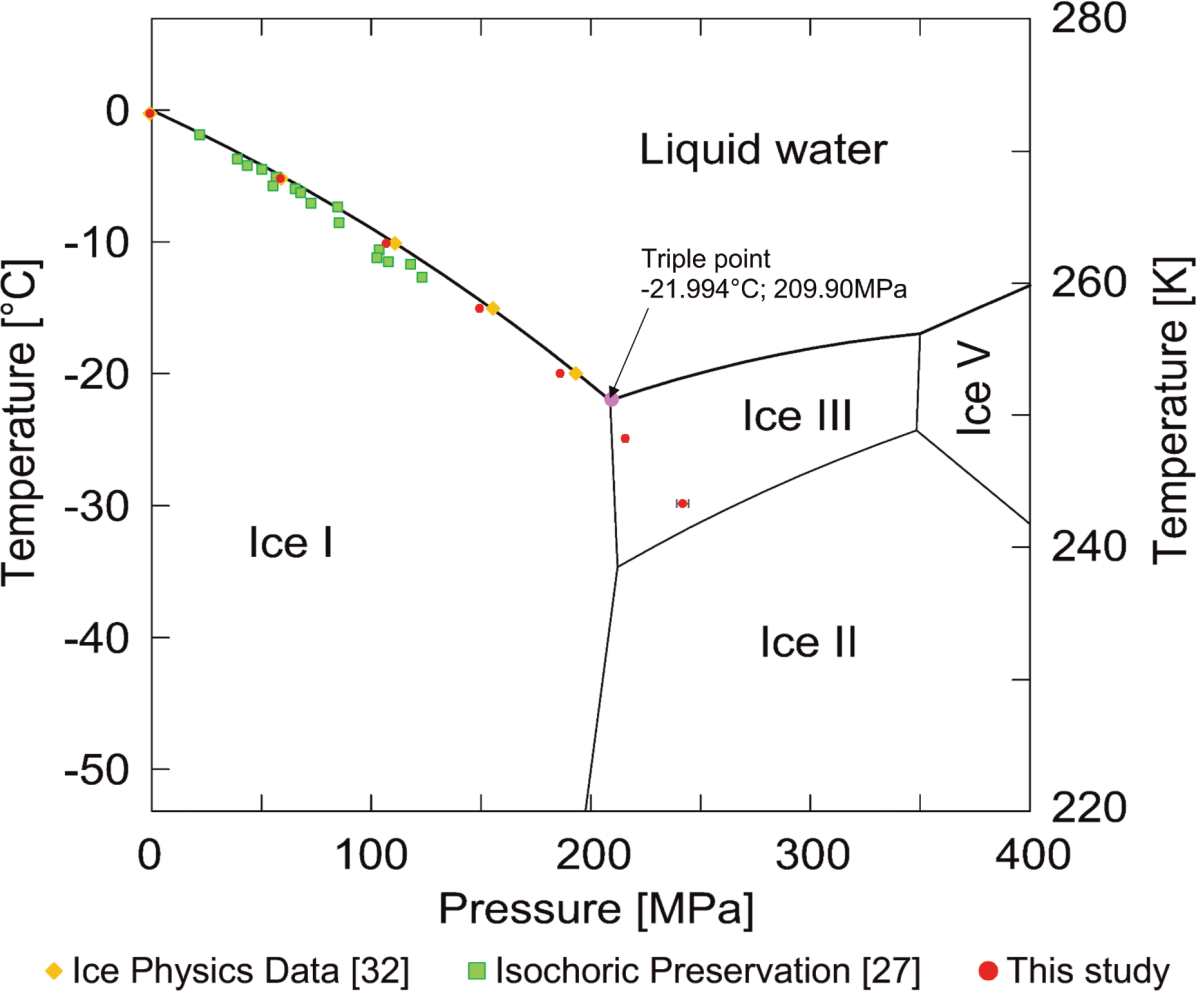
Comparison of experimentally determined pressure and temperature data from this study, with data from other publications, overlaid on a phase diagram for pure water (with modification from [38].

Fig 3 displays the pressure/temperature data during freezing to– 30°C, overlaid on the phase diagram, in comparison to the data from two references, [27, 32]. One of the references is a previous study, with a different isochoric device [27]. The results should not depend on the volume of the isochoric device. The results of this study conform well with the data from those references, which increases confidence in the measurements and in the new device used in this study. Measurements beyond the triple point are new. To the best of our knowledge, these types of measurements were not made before, in the context of cryobiology. Fig 3 shows that the pressure keeps increasing for temperatures lower than the triple point. This was unexpected, because ice III and ice II have a higher density than ice I, therefore, the pressure was supposed to decrease. It is evident that all the data points to the triple point–fall on the liquidus line. However, the data points at temperatures below the triple point are at higher pressure than the triple point. Thermodynamic equilibrium would predict that as the temperature is dropped further, the temperature pressure line would follow the ice I–ice III boundary. However, it does not. A possible explanation is the formation of a metastable form of ice I—ice III mixture. Obviously this observation requires further in-depth research.

**Fig 4:**
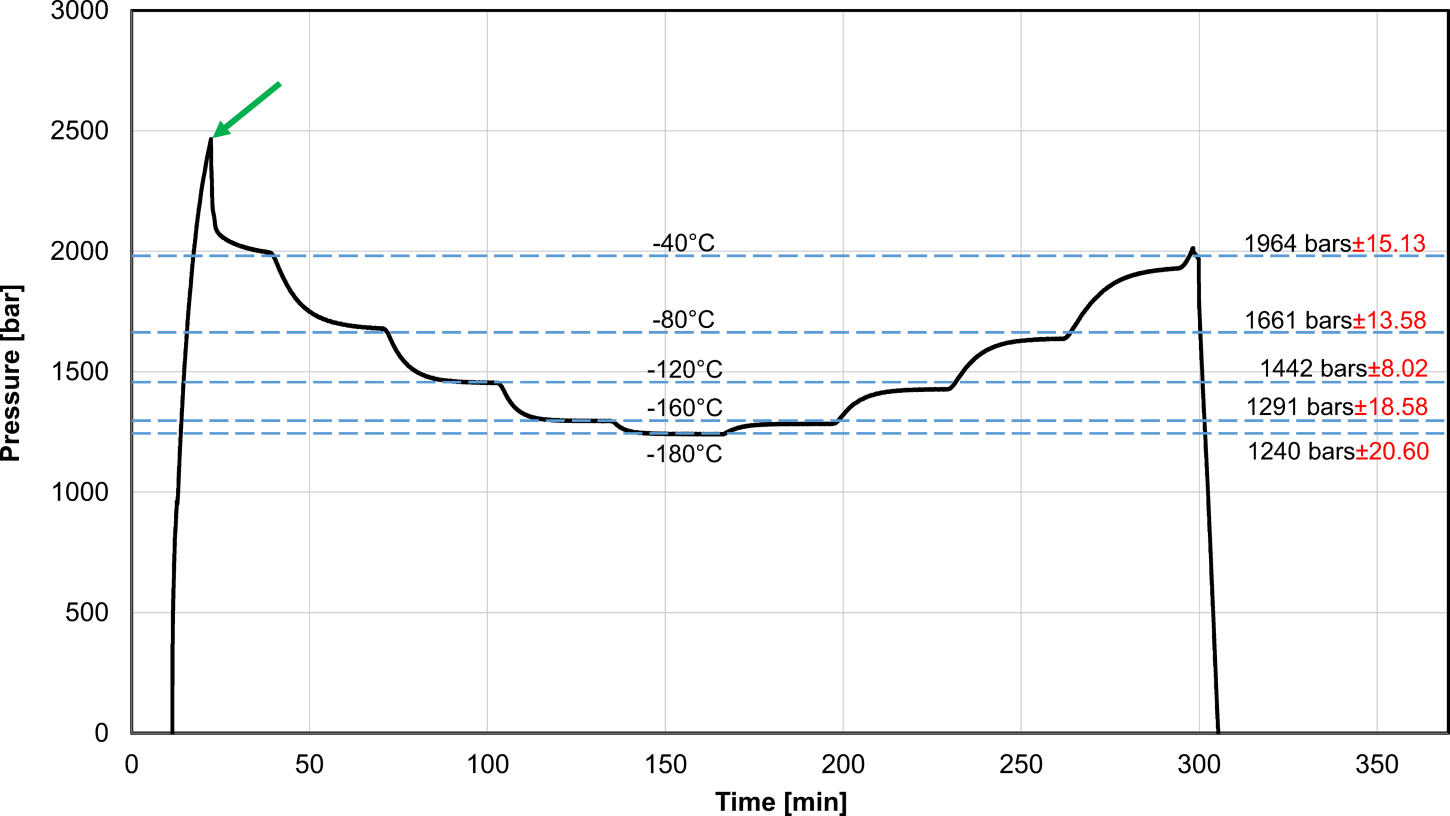
Pressure as a function of time during cooling and warming. The experiments were performed by setting a constant temperature for periods of 30 minutes during cooling and warming. The constant temperatures and their corresponding steady state pressure (with the standard deviation) measured are listed on the figure. The arrow points to the spike in pressure during cooling to– 40°C. Supporting information contained in S2 File

Fig 4, shows typical results from the second set of experiments with pure water, in the temperature range from– 40°C to– 180°C. The results in this figure are typical to all three repeats. The data in Fig 4 is presented in a similar manner as the data in Fig 2. Fig 4 displays pressure as a function of time throughout the experiments. The temperature was changed stepwise and the constant temperature values used in this experiment are listed on the horizontal lines in the figure. Each temperature was kept constant for 30 minutes during both cooling and heating. The results should be read as follows: the intersection between the pressure-time curve and the horizontal temperature lines delineates regions of constant temperatures. For example, during cooling, the part of the pressure-time curve between the– 40°C and the– 80°C, is for the part of the experiment in which the temperature of the Planer device was lowered from– 40°C to– 80°C. However, during heating, the part of the pressure-time curve between the– 40°C and the– 80°C, is for the part of the experiment in which the temperature of the bath was increased from– 80°C to– 40°C. Also, for the segment to and from– 40°C, the base temperature was 20°C. The figure also lists, next to the constant temperature line, the pressures recorded at that temperature at steady state and the standard deviation from six measurements–three during cooling and three during warming.

As from Fig 2, several interesting observations emerge from Fig 4. First, as the system is cooled towards—40°C, a spike in pressure to about 250 MPa occurs. This spike in pressure has occurred in all our experiments. An arrow in Fig 4 points to the spike. The values of pressure at the tip of the spike, are similar to the values measured in the first set of experiments, to– 30°C. This indicates that the increase in pressure at temperatures lower than the triple point, is a genuine phenomenon. However, at– 40°C, the pressure drops. The pressure keeps dropping at each of the measured temperatures to– 180°C. As the temperatures were reduced and then increased, the system was maintained at preselected constant temperatures for 30 minutes, to facilitate thermodynamic equilibrium at those temperatures and pressures. Other interesting observations emerge from this figure. It is evident that steady state values of pressure and temperature are achieved after the 30 minutes at a certain temperature. This suggest that the measurements are for a state of thermodynamic equilibrium. The other interesting observation is that the same values of pressures were obtained at the preselected constant temperatures both while cooling and while heating to those temperatures, i.e. there was no hysteresis. This further supports the suggestion that the measured values were for a system in thermodynamic equilibrium.

**Fig 5:**
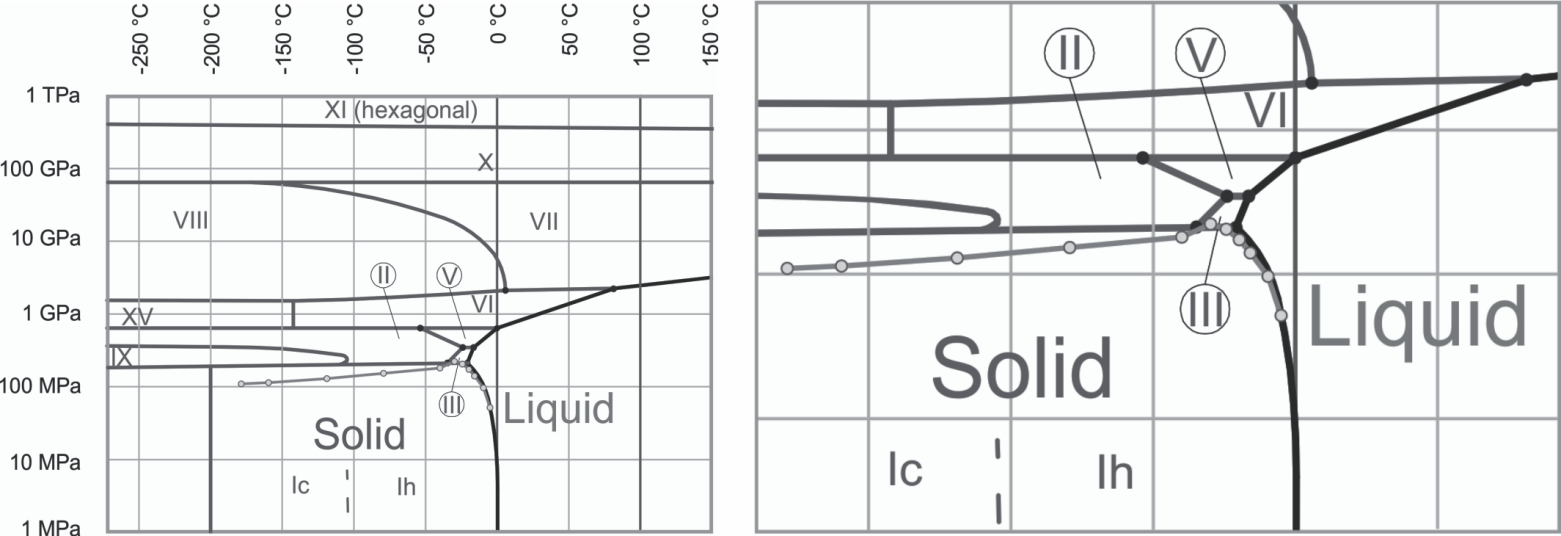
The data from Fig 5 displayed on a phase diagram. The data points–the open circles. The phase diagram outline is a modified version of the phase diagram in [38]

The significance of the measurements in Fig 4 can be better understood when plotted on a phase diagram, as in Fig 5. The error bars in the measurement are small and therefore not visible on the scale of Fig 5, but can be seen in Fig 3 (the most visible is the last value). The thermodynamic state of the system is completely specified by the temperature and pressure (regardless of the volume). Fig 5 shows that the ice that forms is of type I, first hexagonal and then cubic. This information could be valuable in designing optimal cryopreservation protocols and could be obtained only by measuring both temperature and pressure. The figure also shows that the pressure decreases with a decrease in temperature. This is expected because the density of ice I increases with a decrease in temperature to– 243°C.

**Fig 6:**
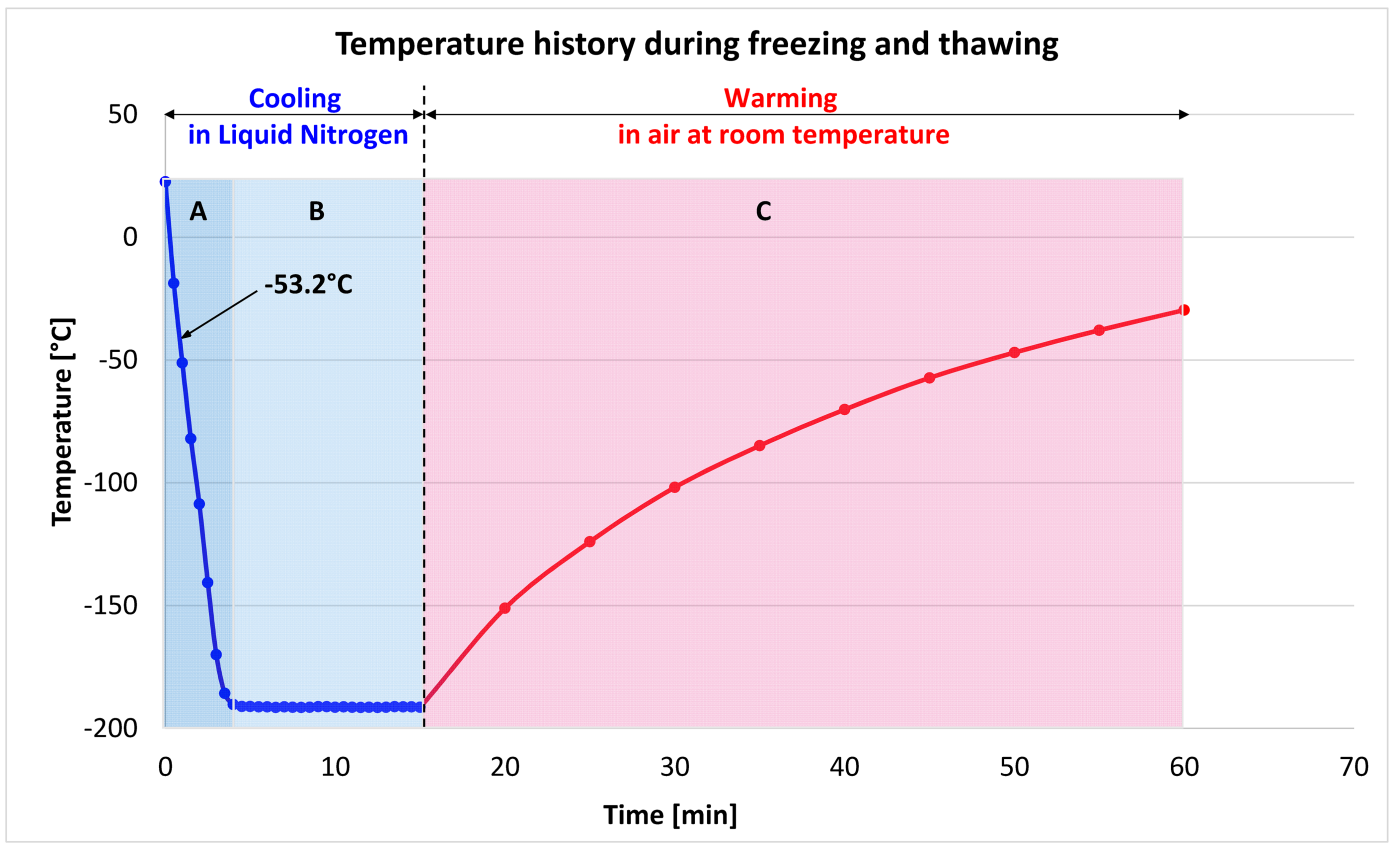
Thermal history during the third set of experiments. Supporting information contained in S3 File

**Fig 7:**
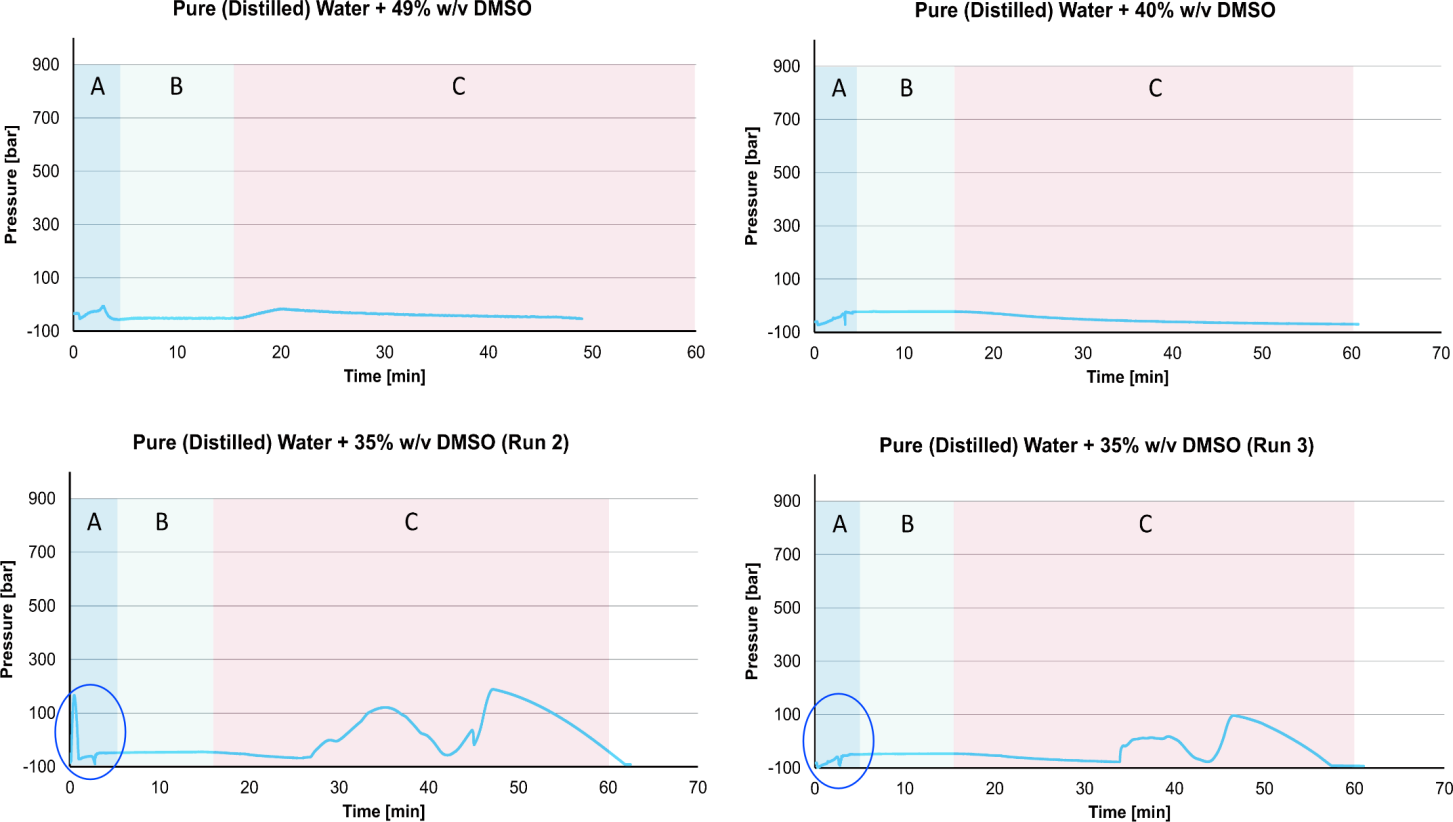
Pressure as a function of time during the freezing and thawing of various solutions of water and Me_2_SO to and from liquid nitrogen temperatures. The three regions of temperature correspond to those marked in Fig 6. Supporting information contained in S4 File

**Fig 8:**
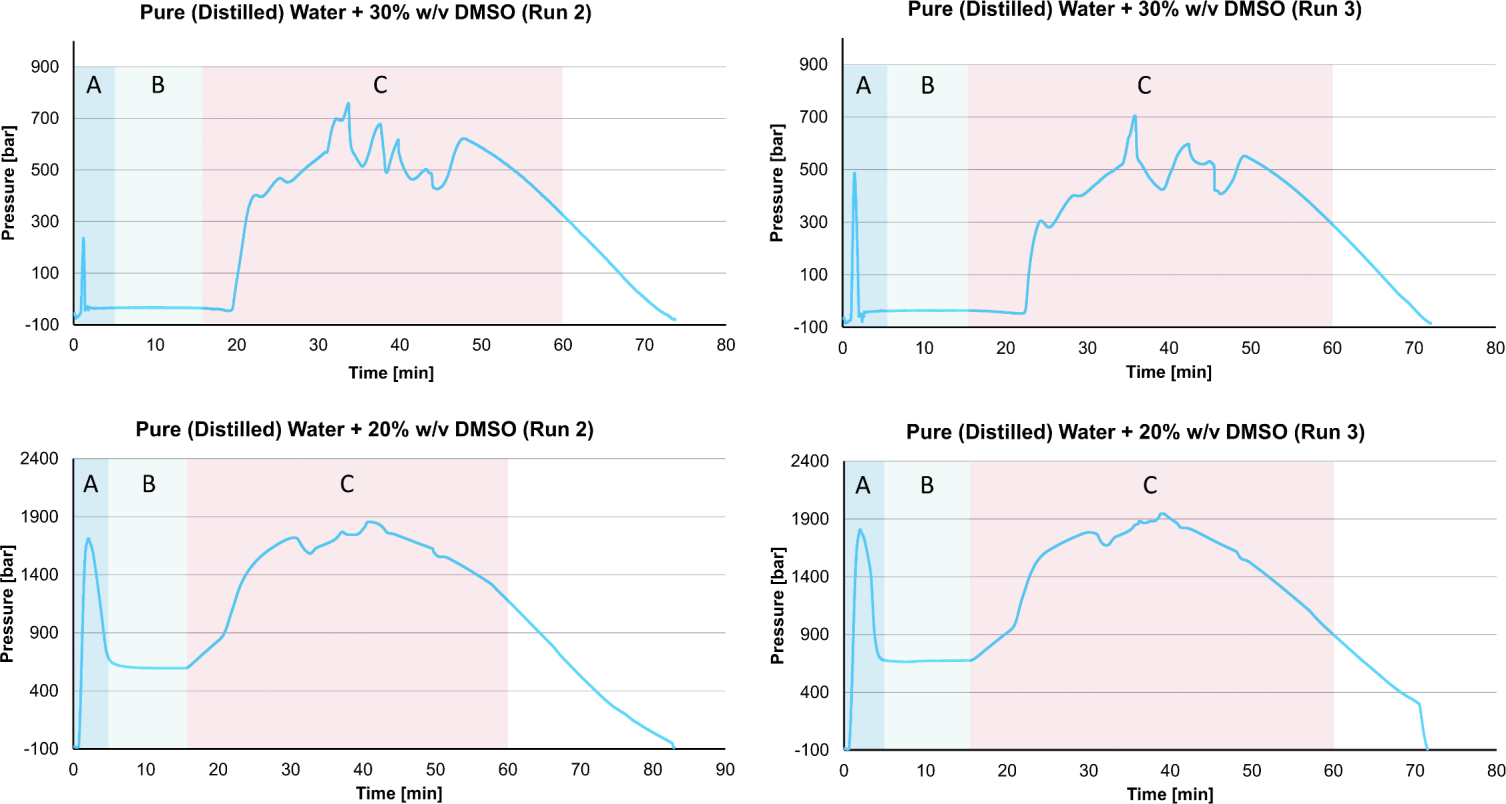
Pressure as a function of time during the freezing and thawing of various solutions of water and Me_2_SO to and from liquid nitrogen temperatures. The temperature history is given in the Fig 6 and the three regions (A, B, and C) correspond to those marked in Fig 6. Supporting information contained in S5 File

The results of the third set of experiments are depicted in Figs 6, 7 and 8. The temperature history during cooling in liquid nitrogen and warming in air, displayed in Fig 6, is particular to the thermal mass of the system used. The cooling and warming rates were not controlled. A similar temperature history was recorded in all runs of the third set of experiments. The thermal regime is made of three regions: A–in which the system cooled to liquid nitrogen temperatures with a cooling rate of– 53.2°C/ min., B–in which the system was maintained at the liquid nitrogen temperature and, C–In which the system was warmed in room air at 22°C.

The primary goal of this experiment was to examine the effect of various concentrations of Me_2_SO on the measured pressure during cooling to and warming from liquid nitrogen temperatures. The concentrations include values known to cause vitrification. Although only select results from the third set of experiments are depicted in Figs 6 and 7, the results are typical to all the repeats unless otherwise stated.

The results in Figs 7 and 8 should be considered in the context of the results in Figs 2 to 5. Figs 2 to 5 show that the formation of ice in the isochoric system is associated with an increase in the pressure measured by the pressure transducer. As a corollary, when there is no measurable change in pressure in the system, at the scale of our measurements, no ice is formed. Therefore, no increase in pressure is expected when a vitrification solution is cooled to liquid nitrogen temperatures.

Fig 7, top left panel shows the pressure as a function of time in a system comprised of pure water and 49% w/v Me_2_SO, during cooling to LN_2_ temperature and warming to room temperature. This composition was chosen because it has been reported, in literature, to produce a vitreous solution (glass–i.e. no ice) when cooled to LN_2_ temperature [10]. The results for 49% of Me_2_SO in pure water, show that there is no increase in pressure neither during cooling nor warming. This is consistent with the assumption above, that the isochoric system measures no discernable increase in pressure when an aqueous solution vitrifies.

Interestingly, the top right panel of Fig 7 shows that there is no increase in pressure during cooling and warming for a solution of 40% w/v ME_2_SO, either. This suggests that ice was not formed in this system, either. Previous studies claim that at this concentration, there should be ice formation under atmospheric pressure [9, 10]. The significance of this observation remains to be further investigated. However, if ice does not form at a concentration of 40% ME_2_SO in an isochoric chamber, in the absence of air, this finding is important for the field of cryopreservation by vitrification. This effect would be consistent with earlier studies. It was found theoretically in [25], that the use of an isochoric system may promote vitrification. While much more research is needed, it appears that the experimental results in this study support the theoretical finding of Szobota [25].

The bottom two panels in Fig 7, were obtained for a concentration of 35% Me_2_SO and show another interesting observation. The bottom left figure shows a spike in pressure during freezing (highlighted by a circle). According to the hypothesis that the formation of ice causes an increase in pressure, this suggests that some portion of the water in the solution has frozen. However, the bottom right panel, which was obtained for a solution with the same composition, shows no spike in pressure (highlighted by a circle). Glass formation, vitrification, is a statistical event. The results suggest that 35% w/v Me_2_SO and the cooling rate used in this experiment are possibly at the margin of the glass formation domain, defined as the intersection between the cooling rate curve and the TTT curve [33]. The two bottom panels in Fig 7 show another interesting observation. The pressure increases during the warming stage of the process. Our hypothesis is that increase in pressure is related to ice formation. The bottom left panel in Fig 7 shows that there is no ice formation during cooling, but ice has formed during warming. This suggests a process of devitrification and ice crystals forming during warming. Fig 7 illustrates the value of measuring pressure in an isochoric system of aqueous solutions designed to undergo vitrification. It suggests that pressure measurements could be used to identify solutions that undergo vitrification and conditions for vitrification and devitrification. This could make the research and application of vitrification for preservation of large biologicals more effective and controlled. Using an isochoric system instrumented with a pressure transducer for vitrification of biological materials could provide real time control over a vitrification based cryopreservation protocol.

Fig 8 is for Me_2_SO concentrations of 30% and 20% w/v. The spikes in pressure during freezing in a solution of 30% Me_2_SO and 20% Me_2_SO, are both evidence that ice crystals have formed in these systems. The substantial increase in pressure during warming, is also evidence that a process of recrystallization has occurred and that low-density ice has formed in the system. The pressure is higher for lower concentrations of Me_2_SO since the amount of water in solutions with lower concentrations of Me_2_SO is higher. This observation is also consistent with the hypothesis that pressure measurements are a valuable means to monitor and control vitrification and devitrification in an aqueous solution in an isochoric system.

The results of this study point to another potentially important observation concerning cryopreservation protocols in “closed volume” systems. Conventional cryopreservation protocols usually monitor and control the temperature history during freezing, storage, and thawing, and the composition of the solution. Usually, a typical description of a cryopreservation protocol contains information on the initial volume and the type of container used. For example, the container is described as a: flexible straw, glass capillary, cryogenic vial, that is capped or uncapped. While in an open volume the pressure is atmospheric and in a hyperbaric system the pressure is set to a constant value above atmospheric, in a “closed volume” system, the pressure may change during cryopreservation in different vessel configuration and materials, e.g. capped versus non-capped, sealed versus non-sealed, glass versus a plastic material. The pressures will also be different in a closed (but not constant) volume with air [34, 35] from those in a closed and constant volume (isochoric) without air [20, 23]. In the first case [34, 35], the range of pressures is 30 psi while in the second case, this study, the pressures are on the order of several hundred MPa. To the best of our knowledge, the literature does not routinely report the monitoring of pressure during cryopreservation in constant volume or closed volume systems. For a randomly chosen example from recent literature, see [36]. That study reports the use of a 0.3 ml CBS™ embryo straws possessing weld seals, i.e. a “closed volume” system. The cooling and warming rates are described precisely, but the pressure is not monitored. However, depending on the rigidity of the walls, the pressure in the system may take different values and, as shown in this paper, the thermodynamic state of the matter in the system may change with pressure. An excellent thorough study on the effects of various “closed volume” container configurations on the outcome of cryopreservation protocols, with an eye towards isochoric cryopreservation is presented in [37]. The authors found better cryopreservation with devices that resemble an isochoric (constant volume) design. However, they also do not measure pressure, which makes their system only partially defined thermodynamically. Consequently, they write: “The process of SPRF (Self-Pressurized Rapid Freezing- a.n.) cryopreservation reaches far deeper (sic) temperatures, but might not be truly isochoric, as faint volume changes of the pressurized tubes due to wall material elasticity could occur.” Measuring only temperature without also measuring pressure and without precise knowledge of the volume does not completely specify the thermodynamic process of cryopreservation in “closed volume” systems.

## Conclusion

The results of this study provide information on the pressure–temperature relationship during isochoric cooling and warming of aqueous solutions to and from liquid nitrogen temperatures. The data may be useful in designing isochoric cryopreservation protocols. The results seem to indicate that real time pressure measurement can identify the occurrence of ice formation during cooling and heating and may serve as a means to study and monitor vitrification and devitrification in isochoric systems of aqueous solutions.

## Supporting information

S1 File Supporting information data for Fig 2. (XLSX)

S2 File Supporting information data for Fig 4. (XLSX)

S3 File Supporting information data for Fig 6. (XLSX)

S4 File Supporting information data for Fig 7. (XLSX)

S5 File Supporting information data for Fig 8. (XLSX)
